# The Evolution of the Sentinel Node Biopsy in Melanoma

**DOI:** 10.3390/life13020489

**Published:** 2023-02-10

**Authors:** Alexandra Allard-Coutu, Victoria Dobson, Erika Schmitz, Hely Shah, Carolyn Nessim

**Affiliations:** 1 Department of General Surgery, University of Ottawa, Ottawa, ON K1N 6N5, Canada; 2 Independent Researcher, Kitchener, ON N6A 3K7, Canada; 3 Department of Medical Oncology, University of Ottawa, Ottawa, ON K1N 6N5, Canada

**Keywords:** melanoma, sentinel node biopsy (SLNB), completion lymph node dissection (CLND), immunotherapy, targeted therapy

## Abstract

The growing repertoire of approved immune-checkpoint inhibitors and targeted therapy has revolutionized the adjuvant treatment of melanoma. While the treatment of primary cutaneous melanoma remains wide local excision (WLE), the management of regional lymph nodes continues to evolve in light of practice-changing clinical trials and dramatically improved adjuvant therapy. With large multicenter studies reporting no benefit in overall survival for completion lymph node dissection (CLND) after a positive sentinel node biopsy (SLNB), controversy remains regarding patient selection and clinical decision-making. This review explores the evolution of the SLNB in cutaneous melanoma in the context of a rapidly changing adjuvant treatment landscape, summarizing the key clinical trials which shaped current practice guidelines.

## 1. Introduction

The pattern of lymphatic spread of cutaneous melanoma is well described [1,2,3]. The sentinel nodes, which are the first lymph nodes involved with disease in a regional nodal basin, have long been recognized as a critical prognostic factor in clinical decision making in the treatment of melanoma. While the treatment of the primary tumour remains resection with wide local excision (WLE), the management of regional lymph nodes in melanoma has evolved significantly over the last 30 years [4,5]. Moreover, with advances in targeted and immune adjuvant therapies, regional lymph node status for accurate prognostication remains highly relevant [6,7]. This article reviews the literature on the evolution of the role of the sentinel node biopsy in melanoma as well as the management of patients with positive sentinel nodes.

## 2. The Discovery of Lymphatics

The ancient Greek philosophers Hippocrates and Aristotle discovered components of the lymphatic system, describing “white blood” and vein-like structures containing a transparent fluid thought to be related to a “lymphatic *temperament*” [1,2,3,8]. In 1650, Swedish anatomist Olaf Rudbeck described a network of closed lymphatic vessels throughout the body, positing on a role of these valved vessels in the etiology of ascites and edema [8]. Coining the term “*vasa lymphatica*”, Bartholin is credited with popularizing the term “lymph fluid” [1,2,3].

In the late 1800s, German pathologist Rudolf Virchow observed that lymph nodes filter particulate matter, and that cancer could metastasize via lymphatic vessels to their corresponding lymph nodes [1,2,3,8,9,10]. This discovery led to the routine dissection of even normal-appearing lymph nodes during the excision of solid malignancies in an attempt to cure cancer before it could spread elsewhere in the body. These findings also inspired William Halstead in his description of the radical mastectomy, with an en bloc axillary dissection, for the treatment of breast cancer in the late 1900s [8,9,10]. Believing lymph nodes to be sieves entrapping cancer cell particulates, he posited they temporarily prevented the systemic dissemination of cancer, thereby creating a window of opportunity for curative surgery via radical node dissections [9,10].

This hypothesis continued to guide the management of solid tumours until the 1960s when the theory of malignancy as a systemic disease by nature gained increasing support, and nodal involvement was hypothesized to be a marker of systemic disease rather than a consequence of an orderly, stepwise progression [1,2,3,8]. The barrier function of lymph nodes was challenged by research involving injecting tumour cells or radiolabelled molecules into afferent lymphatics in animal models [10,11,12]. Fischer and Fischer demonstrated that only 40% of injected stained carcinoma cells were retained by a rabbit’s popliteal node, suggesting lymph nodes were ineffective as barriers to systemic spread [12]. However, the superior prognosis of patients without nodal involvement in several malignancies continued to imply a prognostic role for nodal staging [11].

## 3. First Descriptions of Sentinel Nodes

As early as 1951, frozen sections of the lymph node at the junction between the anterior and posterior facial vein were used to guide the decision to perform a radical neck dissection during parotidectomies [13]. In the 1970s, squamous cell carcinoma of the penis was observed to metastasize to regional lymph nodes without distant metastasis until later in the disease process [1,2,3,13,14]. This led to a report by Dr. Cabañas describing a groin lymph node, frequently the first site of locoregional disease in penile cancer [14]. Studies on the lymphatic drainage of testicular cancer based on the pattern of occurrence of solitary nodal metastasis led Weissbach and Boedefeld to publish an exploration of the feasibility of a limited retroperitoneal lymph node dissection, in which they coined the term “sentinel nodes” and advocated for sentinel node sampling for pathological staging [15,16].

Kett et al. in 1970 injected patent blue dye into the periareolar dermis, visualizing the lymphatic drainage of the breast [17]. They then injected contrast medium into the lymphatics and observed contrast accumulating first into a single lymph node, followed by subsequent drainage to secondary lymphatic channels and nodes in the collecting system surrounding the axillary vein. Similarly, in the 1980s, Christensen et al. redemonstrated primary draining nodes in the axilla using breast lymphoscintigraphy [18]. Dr. Donald Morton at the John Wayne Cancer Center in Santa Monica further innovated the sentinel node biopsy by incorporating lymphatic mapping into the technique. In the 1970s, Dr. Morton first described the use of colloidal gold to identify the lymphatic drainage of melanomas located at ambiguous sites [19]. He later described intraoperative mapping to selectively remove sentinel lymph nodes by injecting patent blue dye intradermally around the primary tumour [20]. Using this technique, lymphatic channels could be visualized intraoperatively and, via careful dissection, followed to the first draining lymph node, which we now call the sentinel node [21].

By the 1990s, the sentinel node biopsy gained popularity as evidence continued to support the existence of an orderly and predictable pattern of lymphatic drainage from primary malignancies to a regional lymph node basin [22,23,24,25,26,27]. The entrapment of tumour cells in the first draining “sentinel” nodes was further supported by a series of 194 lymphadenectomy specimens in patients with a cutaneous melanoma, whereby only two of one hundred and ninety-four had metastatic disease in nonsentinel nodes alone [21]. The sentinel node in this series was involved with a tumour in 38 patients, resulting in a false negative rate of 5% (2/40), confirming that clinically occult nodal metastasis could be accurately identified in early-stage melanoma. Similarly, Turner et al. published a review of 103 breast cancer specimens, revealing that when the sentinel node is tumour free, the probability of involvement of a nonsentinel node is as low as one in one thousand and eighty-seven [26].

For years, regional lymph node dissection routinely accompanied the excision of several solid malignancies. In 2000, the Intergroup Melanoma trial published the long-term results of their study of 740 patients with 1–4 mm thick melanomas. Patients were randomized to have either a wide local excision and immediate elective lymph node dissection vs. wide local excision alone and observation of the nodal basin. There was no difference in overall survival between the two groups (77% vs. 73%) [27]. This lack in an advantage to overall survival was largely due to a very low event rate with a small percentage of the cohort having a positive node in their basin. These findings prompted the hypothesis that there may be a clinical benefit to performing dissections only in patients with positive nodes. This further led to the concept of a sentinel node biopsy to identify which patients have a positive node and thus may benefit from a complete nodal dissection.

As such, with an improved understanding of lymphatic drainage patterns, the sentinel node was defined as the first node in a draining basin to become involved with a tumour, and its status predicted the status of the entire regional lymph node basin [22,23,24,25,26,27]. The validation of intraoperative lymphatic mapping led to the widespread use of the sentinel node biopsy to identify clinically occult disease in early-stage melanoma, sparing patients a regional lymph node dissection without compromising locoregional disease control or staging accuracy, and thus only providing dissections to those that may benefit from it, a form of patient selection for this procedure.

## 4. The Management of Clinically Negative Nodes

Physical examination of regional lymph nodes is often inaccurate for patients with cutaneous melanoma. Despite a clinically negative nodal basin, approximately 20% of patients have occult microscopic metastases on pathologic evaluation of the sentinel nodes [28,29]. Moreover, 20% of patients with clinically positive nodes have pathologically negative basins [29]. Since locoregional disease control in nodal basins is a clinically relevant therapeutic goal in the treatment of melanoma, historically, elective lymph node dissection was offered to all patients with clinically node-negative disease [30,31,32,33]. However, three decades of scientific data have since demonstrated that only 20% of routine completion dissection specimens contained metastatic disease, implying that 80% of patients underwent the procedure without clinical benefit [34,35]. In addition, randomized control trials did not demonstrate improved overall survival for patients undergoing routine elective lymph node dissections, although there may be some survival benefit in certain subgroups as shown in Table 1 [36,37,38].

Evolving technology and improved immunohistochemistry increased the detection of clinically relevant microscopic nodal metastases [39,40,41,42]. The predicted 5-year overall survival ranged from 70% in patients with a single positive SLN with micrometastases to 39% for patients with four or more involved nodes [36,43]. Occult metastatic disease in sentinel nodes has been shown to reliably identify patients at higher risk of recurrence and predicts prognosis [21,30,31,32,33]. In 2009, the American Joint Committee on Cancer staging system recognized the prognostic value of micrometastasis to regional lymph nodes and denoted this stage IIIA disease [40]. The sentinel lymph node biopsy became the standard of care for evaluating clinically negative regional nodes in patients with cutaneous melanoma [30,31,39,40].

Controversy existed with regards to the offering of adjuvant systemic therapy to this heterogenous stage, especially given the historical data placing this stage beneath high-risk stage IIB-IIC disease [40]. A recent retrospective review of 3607 stage IIIA patients suggested adjuvant systemic be offered to all patient with deposits >0.3 mm micrometastasis given at 14.3% calculated absolute DSS difference compared to deposits <0.3 mm [40]. This size cutoff is in keeping with the results from Egger et al. [43] and are in contrast with historically reported <1 mm size cut off deemed to be comparable to sentinel node negative disease [44,45].

### 4.1. Lymphatic Mapping

Sentinel lymph node biopsy (SLNB) is a minimally invasive surgical procedure performed in tandem with the wide local excision of the primary tumour [21]. Lymphatic mapping is used to identify sentinel nodes in the regional nodal basin associated with a malignancy, and these nodes are excised for pathological analysis [21,22,23,24,25]. While cutaneous lymphatic drainage has been shown to follow predictable routes to specific regional nodes, the specific node which first receives lymphatic flow (i.e., the sentinel node) is difficult to determine using anatomic imaging techniques [46,47].

Lymphoscintigraphy has been shown to be the most accurate method of lymphatic mapping [3,4]. The intradermal injection of a radioactive tracer within 1.5 cm of a primary lesion identifies the location of the sentinel node(s) preoperatively with a gamma scintillation camera [48]. The surgeon then uses a handheld gamma probe for intraoperative localization [25,46,47,48,49,50,51,52]. Several tracers have been validated for sentinel node scintigraphy, including sulfur colloid and tilmanocept, which are radiolabeled with the gamma-emitting isotope technetium-99m (99mTc) [53]. Sulfur colloid enters lymphatic channels, where it is phagocytosed by the reticuloendothelial system and sequestered into nodal tissue [54]. It is typically filtered through a 0.22-micron filter prior to injection to optimize the particle size and increase uptake into the lymphatics [52,53,54]. Similarly, tilmanocept is a mannose derivative that binds to receptors on macrophages and dendritic cells, which ultimately travel to and are retained within regional lymph nodes [53]. The tracer is detected in nodal tissue receiving lymphatic flow from the injection site within 10 to 30 min of injection, and the half-life of 99mTc is approximately 6 h [53].

Lymphoscintigraphy mapping images should be available for the surgeon to review prior to surgery to facilitate preoperative planning regarding equipment, patient positioning, and expected procedure length [29,30,31,55]. The focal uptake of the tracer can be imaged with a standard gamma scintillation camera, single-photon emission computed tomography (SPECT) or with SPECT plus concurrent CT (SPECT/CT) if a more precise localization is required [56,57]. In addition, gamma scintillation flow images can provide information about the timing and pattern of tracer accumulation to guide the differentiation between sentinel and secondary nodes [58].

As many as 58% of patients will have sentinel lymph nodes (SLNs) in more than one nodal basin [59,60]. In areas of ambiguous and possibly multiple draining nodal basins such as the trunk, head and neck, and distal extremities, all nodal basins which could drain the site of the primary lesion must be imaged [33,61,62]. The tissue between the primary lesion and sentinel nodes should also be examined for uptake of tracer in in-transit nodes [62]. Lymphatic drainage to a contralateral nodal basin is unusual but should be considered, and unusual patterns of tracer uptake must be promptly communicated to the surgeon [33]. Moreover, despite evidence that surgery can be safely delayed for up to 24 h following tracer injection without compromising outcomes [63], the inevitable radioactive decay of the tracer, as well as the sequential movement of tracer through the SLN towards secondary nodes, may decrease the accuracy of nodal staging [54,55,56]. When the tracer injection is scheduled for the day before surgery, increasing the dose and pairing an afternoon tracer injection with an early operating time can lessen the impact of radioactive decay of the tracer [63].

In early studies, the sentinel lymph node was identified in 75–90% of cases, while in the contemporary literature, a sentinel node is identified in 97% of patients [1,2,3,4,46,47]. Poor tracer injection technique, a failure to scan all involved nodal basins, and missed in-transit nodes increase the false negative rate of the sentinel lymph node biopsy [46,47,64,65]. In addition, when sentinel nodal tissue is entirely replaced by neoplastic cells, it no longer sequesters radiolabelled tracers, decreasing the sensitivity and accuracy of the SLNB [4,46,47,64]. Moreover, prior wide local excision of the primary lesion can change the patterns of lymphatic drainage and SLN scintigraphy has only been validated in patients with prior local biopsy or focal excision of the primary lesion [47].

### 4.2. Validation of the Sentinel Node Biopsy in Literature

The validity and accuracy of SLNB in melanoma are measured in terms of false negatives (FN) and the negative predictive value (NPV) [33,47,48,51,52,53,66,67,68]. The false negative rate is the likelihood that an SLNB fails to identify clinically occult nodal spread to the regional basin as a ratio relative to all regional nodal metastases [66,69]. The false negative ratio (FNR) is calculated by dividing the false negatives (i.e., patients with a negative sentinel node biopsy who develop nodal disease) by the total number of patients who ultimately develop nodal disease [69]. The FNR for sentinel node biopsy in melanoma has been reported to range from 10–20% [66,70,71,72,73,74]. The NPV is calculated as the number of true negatives divided by all negative SLNB [68]. The NPV describes the likelihood of a patient with a negative sentinel node developing a nodal recurrence, whereas a low NPV suggests an increased likelihood of nodal recurrence despite a negative sentinel node. Reported NPVs for SLNB in melanoma range from 94–97% [35,36,37,38,69,70,71,72,73,74]. Importantly, increasing thickness, mitogenicity, and ulceration are associated with increased FNR and low NPV [66,67,68,69,70].

A literature review and meta-analysis by Valsecchi et al. published in 2011 included 25,000 patients across 71 studies and reported a sentinel node biopsy false negative rate of 12.5% [33]. Completion lymph node dissection was performed in a minority of patients with negative SLNB (0.5%), and the incidence of positive nonsentinel lymph nodes despite a negative SLNB was 1–2%. A negative SLNB was associated with a 5% risk of future nodal recurrence. The Sunbelt Melanoma Trial further confirmed the test characteristics for SLNB in 2010 [74], where 59 patients (10.8%) were discovered to have a late regional nodal recurrence out of 486 patients with a positive SLNB. This led to the calculation of an overall FNR of 11% and an NPV of 97% for that population [66,74]. Similarly, the Italian multi-institutional prospective study (SOLISM-IMI, Studio Osservazionale Linfonodo Sentinella Melanoma-Italian Melanoma Intergroup) showed an FNR of 14.4% in their patient population of 1313 consecutive patients with primary cutaneous melanoma (Breslow thickness, >1.0 mm or <1.0 mm but with ulceration, Clark level IV–V) [75].

In the Multicenter Selective Lymphadenectomy Trial-1 (MSLT-I), a 3.4% nodal recurrence rate in patients with negative SLNB was described [35,36,37,38]. Gershenwald et al. at MD Anderson Cancer Center reviewed a series of 243 patients with a negative SLNB [64]. Of these, 10 patients (4.1%) with histologically negative sentinel lymph nodes subsequently developed nodal recurrence in the previously mapped basin. Interestingly, re-examination of the original SLNB specimen using serial sections and immunohistochemical staining identified melanoma in the sentinel nodes in eight of ten patients [64]. The authors concluded that immunohistochemical staining for the melanoma markers S100, HMB45, and Melan-A/Mart-1 enhances the sensitivity of the SLNB tenfold, allowing for the detection of one melanoma cell in one hundred thousand compared with one in ten thousand cells with routine stains.

### 4.3. Multicenter Selective Lymphadenectomy Trial-I (MSLT-I)

The Multicenter Selective Lymphadenectomy Trial-1 (MSLT-1) remains the largest published randomized controlled trial evaluating the role of the SLNB in cutaneous melanoma [35,36,37,38]. Patients were randomized to WLE and lymphatic mapping with SLNB (n = 770) versus WLE alone and nodal observation (n = 500) between 1994 and 2002. Patients assigned to SLNB underwent immediate completion lymphadenectomy if a histologically positive node was identified. Patients with a negative SLNB were observed and underwent completion dissection if subsequent nodal recurrence occurred. Individuals assigned to observation underwent therapeutic lymphadenectomy for nodal recurrence.

The 10-year follow-up data were published in 2014 [37]. The primary study group was defined as patients with intermediate-thickness melanomas (1.2 to 3.5 mm, n = 1347); as it was hypothesized, they would be most likely to benefit from SLNB [34]. The cumulative incidence of regional lymph node involvement in patients with intermediate-thickness melanoma was similar in the two groups [37]. Among the 770 patients assigned to lymphatic mapping with SLNB, a positive lymph node was found in 122 of 765 (16.0%). An additional 4.8% subsequently relapsed in the regional lymph nodes, and the overall incidence of nodal involvement was 20.8%. Among the 500 patients assigned to observation, 87 (17.4%) had a clinical relapse in regional lymph nodes at a median of 19 months after randomization.

Across the entire cohort of patients with intermediate-thickness melanoma assigned to WLE and SLNB, the difference in melanoma-specific survival (MSS) was not statistically significant compared with those randomized to WLE alone and nodal observation (81.4 versus 78.3%) (HR 0.84, 95% CI 0.64–1.09). However, the 10-year MSS was significantly improved in patients with positive nodes on SLNB followed by immediate completion dissection compared to patients assigned to nodal observation followed by treatment at the time of nodal recurrence (MSS 62.1 vs. 41.5%; HR 0.56, 95% CI 0.37–0.84). The 10-year distant disease-free survival rate was also significantly improved in this subgroup (54.8 vs. 35.6%, HR 0.62 95% CI 0.42–0.91). This suggested there may be a survival advantage for only those patients that were sentinel node positive, however this was largely criticized as this was a subgroup analysis of a much smaller cohort and so the survival advantage remained unclear which led to the development of the MSLT-2 trial which we discuss shortly.

The study included 311 patients with thick melanomas (>3.50 mm) in their per protocol analysis. Lymph node metastases were identified in 32.9% of patients with thick melanomas at SLNB. An additional 12 patients, who were initially SLNB negative, subsequently were found to have nodal disease. The estimated incidence of disease at 10 years was 42%. There was no difference in 10-year MSS between the two treatment arms, which was 58.9% for the WLE and SLNB cohort vs. 64.4% with WLE and nodal observation (HR 1.12, 95% CI 0.76–1.67). For thick melanoma, the 10-year MSS was not significantly different in those who underwent SLNB and had a positive sentinel node followed by immediate completion dissection compared with those initially managed with nodal observation until found to have nodal recurrence (48.0 vs. 45.8%; HR 0.92, 95% CI 0.53–1.60). There was no significant difference in distant disease-free survival between groups (45.3 vs. 43.8%, HR 0.96, 95% CI 0.56–1.64).

### 4.4. The Sentinel Node in Melanoma–Therapeutic?

A recent article published in 2022 by members of the Multicenter Selective Lymphadenectomy Trials Study Group describes a subgroup analysis of the observation arm from MSLT-II with the aim of determining the effect of SLNB on long-term regional nodal disease control [76,77]. MSLT-II is the largest randomized clinical trial which compared nodal observation with ultrasound vs. immediate completion dissection for patients with a positive sentinel node. There was no overall survival benefit to completion dissection in this study thus eliciting the hypothesis that removal of the sentinel node alone could provide benefit. This study had a prospective long-term follow up of regional basins in patients with melanoma who underwent nodal observation after a positive SLNB [35,36,38]. Among 855 observed basins following a positive SLNB, at 10 years, 80.2% of the basins remained free of recurrence [38,76]. This study population was characterized by predominantly intermediate thickness melanoma (1.5–3.5 mm, 49.3%) and thick melanoma (>3.50 mm, 24.9%). The median Breslow depth was 2.20 mm and most were nonulcerated (n = 495, 60.2%). The majority were found to have one (n = 699, 81.8%) or two (n-141, 16.5%) positive SLN in the axilla (n = 433, 50.6%) or the groin (n = 316, 37%) at the time of the original SLNB the majority of which had a metastatic deposit <1 mm in size.

A total of 148 (17.3%) nodal recurrences were reported in the 855 basins over 10 years, with a mean 3-year RFS rate of 83.2% and a mean 10-year RFS of 80.2%. SLN basin disease-free survival rates were 90.5% at 5 years and 80.2% at 10 years. By multivariable analysis, younger age (HR 0.57; 95%CI, 0.39–0.84; *p* = 0.004), thinner primary (HR 0.40; 95%CI, 0.22–0.70; *p* = 0.002), axillary basin (HR 0.55; 95%CI, 0.31–0.96; *p* = 003) and an SLN metastasis diameter of less than 1 mm (HR 0.52; 95%CI, 0.33–0.81; *p* = 0.007) were associated with improved nodal basin control [76]. The authors conclude that these findings support a possible therapeutic effect of SLNB in providing long-term locoregional control in over 80% of patients with SLN metastases, sparing them the morbidity associated with a completion dissection.

Despite this well-documented improvement in recurrence-free survival after SLNB for patients with both intermediate-thickness and thick melanomas, the clinical relevance remains unclear. Firstly, the difference in recurrence-free survival and regional nodal basin control is primarily driven by an expected initial nodal failure in the observation arm. There are increased complications after lymph node dissection for clinically positive nodes at the time of a nodal recurrence compared to CLND after a positive sentinel node, as well increased rates of nodal basin relapse after therapeutic LND compared to completion LND. However, these events are relevant only to a small subset of patients. To date, there is no data comparing complications following therapeutic LND or the loss of locoregional disease control with the number of patients who experienced complications following an SLNB. In addition, the trials studying the benefit of SLNB in melanoma predate the availability of effective immunotherapy. Indeed, only 6.5% of patients in MSLT-II received adjuvant therapy, which would have likely further improved nodal recurrence rates and clinically relevant outcomes [77,78].

### 4.5. Criticisms and Considerations

The literature suggests one in five patients with an intermediate thickness melanoma will have a positive SLNB, and thus four of five will undergo the procedure unnecessarily [35,36,37,38]. For thinner melanomas (0.76–2.00 mm), only 2–10% will have a positive SLN [38]. In the absence of definitive evidence that removal of occult disease in the lymph nodes improves survival, hematogenous or distant seeding of tumour cells may occur too early in the disease process for the SLNB to be therapeutic [78]. Indeed, subsequent completion dissection after a positive SLNB has not been shown to improve disease-free survival when nodal relapse is excluded [76,77,78,79,80,81].

Moreover, observation of the nodal basins may allow for nodal micrometastasis to enlarge and spread [76]. In MSLT-I, the SLN micrometastasis incidence was 15.8%, and nodal relapse in the observation group was 16% [35,36,37]. However, in the SLNB group, the median number of involved nodes was 1.4 versus 3.3 at nodal relapse (*p* = 0.0004). How this data translates into clinically meaningful metrics such as risk of distant disease or overall survival remains unclear.

MSLT-I also suggests a survival advantage for the node-positive patients in the intervention arm compared to the observational arm, especially for intermediate thickness melanoma. However, this subgroup analysis is not an intention-to-treat analysis and excludes the falsely node-negative patients in the SLNB arm with subsequent nodal relapse. There were also patients in the intervention arm with negative SLNB who developed distant disease, therefore prognostic false negatives, which were also excluded from this analysis. Other authors describe additional sources of bias from this analysis, including the younger median age of the patients in the intervention arm, which is historically associated with a higher incidence of positive nodes and better overall outcomes [78,79,80,81].

It is conceptually challenging to compare the survival of patients with positive SLNB to patients who recurred with nodal disease after a period of observation. This comparison assumes all abnormal cells identified in the sentinel nodes would become clinically important diseases. In fact, many abnormal cells identified in nodal tissue are destroyed by the immune system and the harsh environment of the lymphatic system and bloodstream. Theoretically, a subset of these positive SLNs is falsely positive [82,83]. Progression to clinically relevant and/or palpable nodal disease might not have occurred even if those positive sentinel nodes had not been removed. This subset of false positive patients is thus incorrectly upstaged, and unnecessarily undergoes further surgery and/or adjuvant therapy in the absence of any proven clinical benefit [79,80,81].

### 4.6. Risks and Complications

The decision to pursue SLNB is based on an individualized discussion of risks and benefits for each patient, as well as the predicted risk of SLNB positivity. In general, the risks associated with SLNB are low, including seroma, wound infection, and, rarely, lymphedema as shown in Table 1 [35,36,37,38,65,84,85]. MSLT-I and the Sunbelt Melanoma Trial study demonstrated that SLNB is associated with significantly fewer complications than regional lymphadenectomy [35,36,37,38,84,85]. At a median follow-up of 16 months, the overall complication rate was lower for SLNB than for CLND (5% vs. 23%), with a lower incidence of wound infections (1% vs. 7%), seroma (2% vs. 6%), sensory nerve injury (0.2% vs. 1.8%), and lymphedema (0.7% vs. 11.7%) [35,36,37,38]. This was especially true for groin procedures, where the overall complications rate was 8% following SLNB compared to 51% after CLND, and where the incidence of lymphedema after SLNB was 2% compared to 32% after CLND [35,38]. The radiation dose associated with lymphoscintigraphy is trivial [3,46,51]. Very small amounts of radioactivity are injected, and much of that activity is removed during surgery. When CT is added to SPECT (SPECT/CT), the CT is typically not performed with contrast, is only of a limited area, and is usually performed with a low-dose imaging protocol, delivering only a fraction of the dose of diagnostic CT scans [56].

**Table 1:** Morbidity of Sentinel Node Biopsy vs. Completion Lymph Node Dissection.

	Total (%)	Lymphedema (%)	Wound Infection (%)	Seroma/Hematoma (%)	Sensory Nerve Injury (%)
SNB [35,36,37,38]	3–8	0.7–1	data	0.5–2	0–0.2
CLND [35,74]	23–51	12–25	data	6–20	3–18
Axilla [74]	12–45	12	data	2–17	4–45
Groin [38,74]	15–72	20–45	data	20–32	15–34

### 4.7. Patient Selection: Current Clinical Guidelines

The threshold at which the prognostic benefits of the SLNB outweigh the procedure’s risk must be evaluated individually, given the evolving role of the SLNB in melanoma. A predicted SLNB positivity rate greater than or equal to 10% is a reasonable threshold, below which the procedure is not recommended [30,34,35,36,37,38,39,40,81]. As the incidence of nodal metastasis has been shown to vary by primary melanoma thickness, the depth of the primary tumour guides patient selection for SLNB in melanoma, in addition to other high-risk features on pathology (Table 2) [1,2,3,35,36,37,38,39,40,50,86,87,88,89,90,91].

### 4.8. Thin Melanoma (<1-mm Breslow Thickness)

The overall risk of SLNB positivity in this subgroup is approximately 5%, and therefore routine SLNB is not recommended for T1a melanomas (nonulcerated, <0.8 mm) [30,33,34,35,36,37,38,81,82]. It may be reasonable to offer SLNB when multiple risk factors are associated with the patient or the primary lesion, such as lymphovascular invasion (LVI) and a high mitotic rate, which increase the predicted SLN positivity to 10% [30,33,78,83,84,85,86]. For T1b lesions (0.8–1 mm +/− ulceration, <0.8 mm with ulceration), SLNB should be considered, given that the predicted SLN positivity is at least 5–10% [81,86,87,88,89].

### 4.9. Intermediate-Thickness Melanoma (>1 mm to 4 mm Breslow Thickness)

SLNB in this population is strongly supported by the MSLT-I trial, where the estimated risk of SLNB positivity approached 15% [35,36,37,38,39,89,90,91]. Given the prognostic significance and possible therapeutic benefit, SLN biopsy has been widely adopted in intermediate thickness melanoma, which includes patients with T2 or T3 disease [89,90,91]. The SLNB positivity in intermediate thickness melanoma is influenced by several risk factors and is highly variable. Multivariate analysis has demonstrated that tumour thickness (1.00–1.49 mm vs. 1.50–4.00 mm), LVI, tumour-infiltrating lymphocytes, and microsatellites predict sentinel node positivity in this population [33,34,35,36,37,38,39,40].

### 4.10. Thick Melanoma (>4-mm Breslow Thickness)

Thick melanomas are associated with an increased incidence of distant disease at the time of diagnosis. As such, despite an SLNB positivity that approaches 30–40%, routine SLNB in this subgroup is controversial [35,36,37,38,39,40,81,92]. MSLT-I confirmed SLNB is highly prognostically relevant in this subgroup, where 10-year survival was 65% in patients with a negative SLN vs. 48% in those with a positive SLN; (*p* = 0.003) [35,36,37,38]. Unlike patients with intermediate-thickness melanoma, the subgroup analysis did not suggest a survival benefit with SLNB in this patient population [35]. However, early intervention decreases the morbidity of LND and improves locoregional control, especially for thicker melanomas where locoregional disease is more common [35,36,37,38,76,78]. In the era of increasing effectiveness and availability of adjuvant therapy for melanoma, the status of the SLN is increasingly relevant as it may confer eligibility to certain treatments and/or clinical trials [92].

**Table 2:** Special Considerations.

Prior WLE [81,93]	Disrupts lymphatic drainage and could limit accuracy of the SNB. Retrospective data support the prognostic value of SNB post prior WLE. Significant flap reconstruction is associated with an increased false-negative rate.
Isolated Local Recurrence [93,94,95,96]	Consider SNB for isolate recurrence or limited in-transit disease if nodal status informs a decision for adjuvant therapy or eligibility clinical trials. The rate of positive SNB up to 40%, and nonsentinel node up to 30–40%. It remains unclear if CLND is prognostic or therapeutic in this population
Pregnancy [39,40,81]	No absolute contraindications to melanoma surgery during pregnancy. Radioactive colloid tracers are considered safe. Isosulfan blue contraindicated due to rare risk of severe allergic reactions. Methylene blue contraindicated as it causes developmental malformations. Treatment decisions require multidisciplinary team.
Microsatellites [81,95,96]	Heterogeneity in OS in this patient population. In the absence of negative prognostic features (i.e., ulceration, positive nodes), some patients with overall good prognosis may benefit from SNB. Consider SNB regardless of thickness of the primary if it will guide treatment.

## 5. Management of Positive Sentinel Nodes

Sentinel nodes are examined by serial sectioning followed by methodical pathological analysis, including immunohistochemistry and melanoma-specific stains [4,5,15,16,36,86,97,98,99,100]. Fewer nodes are examined in greater detail, increasing the detection of smaller metastasis [97,98]. The positivity of nonsentinel nodes was 8.4% higher in the observation arm of MSLT-II, which underwent nodal observation after a positive sentinel node, suggesting completion dissection may miss as many as two of five nonsentinel node metastases [38]. It also suggests that node positivity following completion dissection may underestimate the burden of nonsentinel node metastasis.

Moreover, despite evidence that a low volume of tumour in a sentinel node decreases the probability of metastatic disease in nonsentinel nodes, even minimal metastasis to sentinel nodes increases the risk of locoregional recurrence and death relative to patients with negative sentinel nodes [100]. Bremmer et al. reviewed a database of 1250 consecutive patients who underwent SLNB for primary cutaneous melanoma [97]. The 5-year MSS of patients with melanoma cells confined to intracapsular lymph vessels, subcapsular or transverse sinuses (sentinel node invasion level [SNIL] 1) was comparable to that of node negative patients (91.4%). In contrast, outgrowth of the metastasis from the parenchyma into the fibrous capsule (SNIL2) or the medulla (SNIL3) of the lymph node was associated with a significantly worse prognosis (83.5% and 31.7%, respectively, *p* < 0.0001). SNIL2 and 3 were also associated with an increased 5-year locoregional recurrence (SNIL2—7.8% with CLND vs. 24.8% without; SNIL3—42.3% with CLND vs. 52.4% without).

The prognostic relevance of the extent of tumour volume in sentinel nodes was further demonstrated in a retrospective study by the European Organization for Research and Treatment of Cancer (EORTC) of 1009 patients with a positive sentinel lymph node who underwent completion lymph node dissection [99]. Sentinel node metastasis of less than 0.1 mm were found to have outcomes comparable to those of sentinel node-negative patients, with a 5-year overall survival rate of 91% and a nonsentinel node positivity rate of 9%. Nonsentinel node positivity increased with increasing tumour volume (16% for 0.1–1.0 mm nodal metastasis and 25% for metastasis >1.0 mm) and decreased 5-year overall survival rates (71% for 0.1–1.0 mm nodal metastasis and 57% for metastasis >1.0 mm). These findings suggest that the extent of sentinel node invasion may represent an important prognostic marker which could guide treatment recommendations.

### 5.1. Multicenter Selective Lymphadenectomy Trial-II (MSLT-II)

MSTL-II is a large phase III randomized controlled trial which compared CLND (n = 824) to serial nodal basin observation with ultrasonography for patients with a positive SLNB (n = 931) [35,36,38]. The study included patients with primary melanomas with a Breslow thickness ≥ 1.20 mm or with ulceration regardless of thickness. Notably, while both arms were balanced in terms of patient and tumour characteristics, most patients in this study had low-volume (<1 mm) metastasis deposits in the positive sentinel node [35]. MSS was the primary endpoint and was the same in both study arms (86% for the CLND group vs. 86% in the observation arm at 3 years, adjusted HR 1.08, 95% CI 0.88–1.34) [38]. The improved DFS at three years in the CLND group (68% versus 63%) is felt to reflect regional nodal recurrences in the observation arm (8% versus 23%). No subgroups were identified in whom upfront completion lymphadenectomy provided a clear benefit.

### 5.2. German Dermatologic Oncology Cooperative Group (DeCOG-SLT) Trial

DeCOG-SLT is a multicenter trial that randomized 483 patients with cutaneous melanoma and a positive SLNB to CLND or observation with serial ultrasound examinations of the appropriate lymph node basin [84,85]. Approximately 2/3 of enrolled patients had low-volume disease in the sentinel node. The primary outcome of MSS was comparable between groups (HR 1.19; 90%CI, 0.83 to 1.69; *p* = 0.43), as was OS (HR 1.02; 90% CI, 0.68 to 1.52; *p* = 0.95) and RFS (HR 0.959; 90% CI, 0.70 to 1.31; *p* = 0.83). An expected difference in regional nodal recurrence was observed (16.3% in the observation arm vs. 10.8% post-CLND).

### 5.3. Outcomes after MSLT-2 and DeCOG-SLT

Both MSLT-II and DeCOG-SLT revealed CLND for patients with a positive SLNB does not improve survival when compared with serial ultrasound surveillance (Table 3). For patients with a positive SLNB who do not undergo a CLND, guidelines based on these trials recommend ultrasound surveillance of regional lymph nodes every four months during the first two years, then every six months for up to five years [35,36,37,38,84,85,101]. Nodal surveillance is typically discontinued after five years [38,81]. CLND may be indicated in lieu of nodal surveillance if access to surveillance imaging is logistically challenging, if the primary tumour histology and nodal tumour burden suggest an increased likelihood of nodal involvement and/or recurrence, or when adjuvant therapy will not be pursued [38,81,82,83,101,102,103,104,105,106,107,108,109,110].

Analysis of the National Cancer Database in the US reveals a decline in patients undergoing CLND for melanoma following a positive SLN, with the rate dropping from 55.9% (n = 9725) in the pre-MSLT-II era to 19.5% (n = 9419) post-MSLT-II (odds ratio [OR] 0.32, 95%CI 0.29–0.35) [102,111,112]. Similarly, a retrospective cohort of 1154 SLNB-positive patients treated post-MLST-II at 21 institutions in Australia, Europe, and the United States revealed that, after negative distant staging, 965 patients (84%) received active surveillance while 189 (16%) underwent CLND [113]. Four hundred and thirty-nine patients received adjuvant therapy (38% in the surveillance group and 39% in the CLND group), with the majority (83%) receiving anti-PD1 immunotherapy.

## 6. The Era of Immunotherapy

### 6.1. Overview of Immunotherapy and Targeted Therapies

Advancement in our understanding of the pathobiology of cutaneous melanoma has supported greater therapeutic use of targeted therapy and immunotherapies. Their use has resulted in improved and durable oncologic outcomes as compared to traditional chemotherapy, and consequently has revolutionized melanoma care.

Broadly, immunotherapy leverages the host’s immune system against a malignancy. These therapies largely mediate T cell activation and function by non-specific cell stimulation by cytokines (interferon-α 2b/pegylated interferon-α 2b, IL-2), or promoting T cell activation by antibody blockage of immune checkpoint inhibitors cytotoxic T lymphocyte antigen 4 [108], CTLA-4, ipilimumab, and programmed cell death protein 1 interaction with cell surface ligands-1 and -2 [104], PD-1, pembrolizumab, nivolumab, and gene modifying oncolytic viruses (talimogene laherparepvec [109], T-VEC). Additional mechanisms under study for systemic treatment development include adoptive T cell transfer by infusion of autologous tumor-specific T cells (CAR-T), oncolytic viruses, tumoral vaccines, amongst others summarized in a recent review [104,114].

Molecularly targeted therapy targets mutated proteins implicated in oncogenic signaling pathways. The most relevant cell signaling pathway is the mitogen-activated protein kinase (MAPK) or RAS-RAF-MEK-ERK pathway [105]. The most common mutations of cutaneous melanomas include NRAS of the RAS proteins, BRAFV600E and V600K of the RAF proteins [61,106,112], vemurafenib, dabrafenib, and MEK1 and 2 of the MEK proteins [107,113], cobimetinib, trametinib. A second pathway more rarely implicated in cutaneous melanoma development is the phosphatidylinositol 3-kinase and protein kinase B (PI3K-AKT) pathway. C-KIT cell surface protein receptor activates both the MAPK and PI3K-AKT pathways [102,108], imatinib.

### 6.2. Evolution of Systemic Therapy

Prior to 2015, interferon-α 2b was the only approved agent for adjuvant treatment of patients with high-risk cutaneous melanoma [115,116,117,118]. Interferon-α-2b exhibits anti-tumour activity by enhancing natural-killer cell activity as well as tumour antigen presentation and has been shown to have anti-proliferation and anti-angiogenic effects [115]. Widespread use was limited by a significant side effect profile combined with limited clinical benefit. A marginal improvement in disease recurrence was noted across 17 studies included in a Cochrane systematic review and meta-analysis published in 2013 (HR 0.82 compared to observation, 95%CI 0.78–0.87), without improvement in OS in resected node-positive melanoma [118]. OS was improved in patients with ulcerated primary melanomas (HR 0.77; 95% CI 0.64–0.92) and it is felt that this was driving the clinical benefit noted in earlier studies as this subgroup represented over a third of the study population [115,118]. Subsequently, anti-cytotoxic T-lymphocyte antigen-4 (CTLA-4) and anti-programmed death-1 (PD-1) immune-checkpoint inhibitors as well as mitogen-activated protein kinase-directed (MAPK) targeted therapies were validated in the adjuvant setting, leading to important changes to clinical practice (Table 4) [119,120,121].

### 6.3. Immune-Checkpoint Inhibitors

EORTC 18071 is a randomized phase three clinical trial comparing ipilimumab (anti-CTLA-4) vs. placebo in patients with resected stage III melanoma [122]. Ipilimumab was given at a dose of 10 mg/kg every three weeks for four cycles, followed by maintenance doses every three months for up to three years. The 5-year RFS was 41% in the ipilimumab arm vs. 30% in the placebo arm (HR 0.76; 95%CI 0.64–0.89). Distant metastasis-free survival (DMFS) was also improved in the ipilimumab arm by 48% vs. 39% with placebo (HR 0.76; 95%CI 0.64–0.92), as was overall survival (HR 0.72; 95%CI 0.58–0.88). The treatment was associated with significant toxicity. Grade 3–4 adverse events were observed in over half of the patients in the treatment and five patients died due to immune-related toxicities. This greatly limited the implementation of ipilimumab in the treatment of melanoma despite subsequent studies, such as E1609 which confirmed the efficacy and improved the safety profile at lower doses [115,116,117,118,122].

In advanced disease, the anti-PD-1 agents nivolumab and pembrolizumab were shown to be safer and more effective, and as such, their use was explored in the adjuvant setting [117,119,120,121]. CheckMate-238 is a randomized, double-blind phase three clinical trial randomizing patients with resected stage IIIB, IIIC, and IV melanoma to receive either nivolumab dosed at 3 mg/kg every 2 weeks for a year or ipilimumab 10 mg/kg every 3 weeks for four cycles and then every 12 weeks for up to a year [127]. RFS was reported as 63% at 2 years for patients treated with nivolumab vs. 50% for those treated with ipilimumab (HR 0.66; 95%CI 0.54–0.81). The DMFS at 2 years was also improved with nivolumab (70.5% vs. 64% with ipilimumab). These effects were independent of PD-L1 expression, disease stage, and BRAF mutation status. Nivolumab was associated with less toxicity and severe adverse events were reported in 14% of patients.

Keynote-054 is a randomized, double-blind phase three clinical trial evaluating the use of pembrolizumab in the adjuvant setting for patients with resected stage IIIA (with >1 mm lymph node metastasis), IIIB, and IIIC melanoma [126]. Patients were randomized to receive pembrolizumab at 200 mg every three weeks or a placebo. The trial included a crossover design, whereby documented recurrence rendered patients in the placebo arm eligible for crossover to the treatment arm, and patients previously treated with pembrolizumab could be retreated if the recurrence occurred more than 6 months from the last treatment. Despite the crossover, RFS remained significantly improved with pembrolizumab, with an 18-month RFS of 75% vs. 61% in the placebo arm (HR 0.57; 98%CI 0.43–0.74), as was DMFS (HR 0.53; 99%CI 0.37–0.76). This effect was independent of PD-L1 expression, disease stage, and BRAF mutation status. Serious adverse events were reported in 15% of patients treated with pembrolizumab, including one death from pembrolizumab-related myositis. Keynote-054 continued to demonstrate improved RFS in the 3-year interim analysis (63.7% vs. 44.1%; HR 0.56; 95%CI, 0.47–0.68) but has yet to report OS due to low event rates [117]. CheckMate 238, after 51 months of follow-up, reported a persistent difference in RFS (HR 0.71; 95%CI 0.60–0.86; *p* = 0.0003) without a significant difference in OS across treatment arms [127]. Importantly, the reported 4-year OS was 78% in the nivolumab arm and 77% in the ipilimumab arm (HR 0.87; 95% CI 0.66–1.14; *p* = 0.31), with fewer than expected deaths in both study arms. In addition, 57% of patients (143 of 253) in the ipilimumab arm received subsequent crossover immunotherapy at the time of recurrence.

Both nivolumab and pembrolizumab are approved for the adjuvant treatment of resected stage III melanoma and nivolumab is approved for the treatment of resected stage IV disease [115,116,117,118]. More recently, the results of KEYNOTE-716 led to the approval for adjuvant pembrolizumab for resected high-risk stage II disease as well [119]. This study randomized 976 patients with stage IIB or IIC melanoma to receive pembrolizumab versus placebo. With a median follow-up of 20.5 months, adjuvant pembrolizumab was shown to improve 18-month RFS (HR 0.61; 95% CI 0.45–0.82). Node-negative melanoma is associated with a lower risk of recurrence and death compared to historical populations studied in the context of adjuvant therapy for melanoma, and as such larger cohorts will require prolonged follow-up to identify whether there is a statistically significant OS benefit in patients with stage IIB or IIC melanoma treated with adjuvant pembrolizumab [115,116,117,119].

### 6.4. Targeted Therapy

BRAF is a serine/threonine protein kinase which activates the MAP kinase/ERK-signaling pathway and approximately 50% of cutaneous melanomas are found to have BRAF V600 somatic mutations [115]. The BRAF inhibitors vemurafenib and dabrafenib were the first to be approved for the treatment of BRAF-mutated melanoma [116]. BRIM-8 is a phase three randomized controlled trial which randomized patients with resected high-risk BRAF-mutant melanoma to receive vemurafenib vs. placebo [125]. Enrolled patients were divided into two cohorts. DFS was improved in cohort one, which included resected stage IIC, IIIA (with lymph node metastasis > 1 mm), and IIIB disease (HR 0.54; 95% CI 0.37–0.78). The improvement in DFS did not reach significance in cohort two, which included resected stage IIIC disease (HR 0.80; 95% CI 0.54–1.18), favoured to be due to a low event rate.

Subsequently, randomized phase three trials demonstrated the safety and superior efficacy of combining BRAF and MEK inhibition. COMBI-AD is a phase three randomized controlled trial that randomized 870 patients with BRAF-mutant resected stage III melanoma (with lymph node metastases > 1 mm for stage IIIA) to receive the combination of BRAF inhibitor dabrafenib 150 mg bid and MEK inhibitor trametinib 2 mg daily vs. placebo [123,124]. RFS significantly improved in the treatment arm, where 54% of patients were relapse-free at 4 years vs. 38% in the placebo arm (HR 0.49; 95%CI 0.40–0.59). DMFS was also improved in patients treated with dabrafenib and trametinib vs. placebo (HR 0.53; 95%CI 0.42–0.67). The most commonly reported adverse events included pyrexia (total 63%; grade 3 and 4, 5%), fatigue (47%; 4%), and nausea (40%; 1%). Health-related quality of life (QoL) was not negatively impacted during treatment or long-term follow up [124].

### 6.5. Sentinel Node Biopsy in the Era of Immunotherapy

Adjuvant therapy trials required CLND for sentinel node-positive melanoma before systemic treatment. However, post-MSLT-II, nodal surveillance without CLND has become the standard of care [115,116,117,120,121]. For example, concurrent to MSLT-II, landmark trials led to immune checkpoint blockade and BRAF/MEK inhibitors becoming the standard of care when adjuvant therapy was indicated for resected stage III melanoma in the setting of CLND [121,123,125]. Since adjuvant trials exclusively included patients post-CLND, it was unclear if patients with positive sentinel nodes undergoing active nodal basin surveillance would benefit from similar outcomes [120].

A retrospective cohort of 177 patients with cutaneous melanoma and a positive SLNB managed with nodal surveillance from June 2014 to June 2019 reported outcomes after a median follow up of 24 months [113]. Most patients in this cohort had intermediate thickness tumours (n = 102, 64%) with a single positive sentinel node (n = 135, 76%). The recurrence rate was 27% (n = 48). Of the patients who received adjuvant therapy (n = 66, 37%), 89% received anti-PD1 immunotherapy, 8% received BRAF/MEK inhibitor therapy, and 3% were on clinical trial with combined anti-CTLA4 and anti-PD1 immunotherapy versus anti-PD1 immunotherapy alone. Adjuvant treatment did not alter patterns of initial recurrence (*p* = 0.76) but was associated with increased frequency of nodal ultrasound and cross-sectional imaging surveillance (*p* < 0.01). Most nodal recurrences occurred in the first year.

Moreover, high-risk groups were excluded from MSLT-II and DeCOG-SLT, including patients with microsatellites, extranodal extension in the SN, or >3 positive SNs, which are associated with a 2-fold greater recurrence risk [38,112]. For these patients, uncertainty persists regarding the appropriateness of nodal surveillance. Indeed, over 70% of MSLT-II participants and over 90% of DeCOG-SLT participants had only a single positive SN, with the majority having a single nodal deposit <1 mm in size [35,36,37,38,84,85,111,112]. A cohort of 1154 patients with SLNB-positive cutaneous melanoma treated at 21 international centers in the 2 years after the publication of MSLT-II was included in a retrospective analysis [112]. CLND patients were matched 1:1 with surveillance patients using propensity scores. Among these, one hundred and sixty-six had high-risk characteristics, including fifty-six with extranodal extension, eighty with microsatellites, eleven with more than three positive SNs, and twenty-two with two or more of these high-risk features. At 18.5 months median follow up, 49% had recurrence vs. 26% in patients without high-risk features (*p* < 0.01). Among high-risk patients, 52 (31%) underwent CLND, and 114 (69%) received surveillance. There were no significant differences in recurrence (49% in the CLND arm vs. 45% with surveillance, *p* = 0.9) or melanoma-specific mortality (14% in the CLND arm vs. 12% with surveillance, *p* = 0.86). Most recurrences were outside the SN basin, supporting the use of nodal surveillance. These patients were also more likely to have received adjuvant systemic therapy (51% vs. 42%, *p* > 0.04). The most common adjuvant regimens for this cohort were anti-PD-1 immunotherapy (79%), BRAF/MEK inhibitor therapy (7%), and combination immunotherapy (3%).

As such, the use of adjuvant therapy in patients with a positive SLNB who are being managed by nodal surveillance in lieu of CLND have been shown to have similar rates of isolated nodal recurrences, which remain largely salvageable with therapeutic lymph node dissection [102,111,112,113]. In the era of evolving adjuvant therapy for melanoma, nodal recurrence in the setting of observation after a positive SLNB might represent regional failure due to incomplete clearance of positive nonsentinel nodes and/or treatment-resistance disease. Whether these patients should be re-challenged with the same adjuvant treatment remains unclear.

## 7. Staging

SLNB identifies patients with clinically occult pathologically positive lymph nodes. In patients with a positive SLNB, cross-sectional imaging detects clinically occult distant metastatic disease in 0.5–3.7% of patients [128,129,130,131]. True positive findings are more common in ulcerated thick primary tumours and/or those with increased tumour burden in the sentinel nodes [129]. PET scanning is thought to enhance the detection of subclinical metastatic disease [132,133,134,135,136,137]. A systematic review of 17 studies revealed an overall sensitivity of 68–87% and specificity of 92–98% for PET CT for stage III and IV melanoma, and it may be superior to CT in detecting distant metastases for this population [138]. However, the yield and sensitivity remain low in patients with clinically localized, early-stage disease [132,133,134].

Another consideration for staging imaging at the time of a positive SLNB is the early detection of central nervous system (CNS) metastases. While CNS disease occurs in ≤5% of patients with stage I-IIIB melanoma, it occurs in as many as 11% with stage IIIC disease [81,139,140,141]. Clinically symptomatic CNS metastases are associated with significant morbidity and poor survival, and early detection improves outcomes [139,140]. Similarly, outcomes after treatment of CNS metastasis are significantly improved in patients with lower tumour burden [140]. Thus, baseline CNS imaging should be considered as it may enhance the early detection of CNS metastasis in at-risk patients [81].

## 8. Local Therapies

### Radiation Therapy

Radiation therapy can be a useful modality in the nodal management treatment of cutaneous melanoma. Notably, it has shown to improve local control following lymphadenectomy for stage III high risk patients for nodal relapse (nodes >3 cm, >1 parotid, >2 cervical or axillary, >3 epitrochlear or inguinal, extracapsular extension, and prior recurrence disease) and all patients with nodal recurrence [142,143]. The ANZMTG 01.02/TROG 02.01 phase three randomized control trial compared lymph node field relapse to observation to adjuvant radiation therapy in patients presenting with palpable nodal disease post lymphadenectomy in patients who presented with palpable nodal disease [142].

The authors reported a 15% absolute reduction in nodal recurrence as first relapse, and at any time within the 5-year follow-up period, with no difference in distant recurrence, RFS and OS between both groups. Extranodal extension was an independent predictor of nodal recurrence. Notably, 74% of patients developed radiation-related toxicities, and while lymphedema was seen in both treatment arms, it was reportedly more common amongst patients having undergone extremity lymphadenectomy randomized to the radiation group. Thus, adjuvant therapy has been traditionally reserved for stage III high risk and recurrence nodal disease; the benefits of local control does need be weighed against such risks.

Amongst many mechanisms of action, radiotherapy is associated with decreased local immunosuppression. As a potential area of failure for local control, it is rational to consider combination with immunotherapy to counterbalance this immunosuppressive effect [144]. Combination immunotherapy and radiation therapy has been shown to improve oncologic outcomes in the metastatic and locally advanced setting [145,146,147,148], while adverse reactions have been significant [149,150,151]. No study to date has analyzed combination therapy of nodal disease control in melanoma and this may be an area of study with continuing advances in immuno-oncology [152].

## 9. Conclusions

The management of melanoma is rapidly evolving, incorporating advances in molecular profiling, systemic therapies, and surgical technique. The sentinel node biopsy remains a valuable staging tool in the management of melanoma. In the era of immunotherapy and targeted therapy, the sentinel node biopsy continues to provide important prognostic information. With an improved understanding of the pathogenesis of melanoma and its patterns of dissemination, the sentinel node biopsy may facilitate the identification of patients at higher risk of poor outcomes and guide recommendations for adjuvant therapy.

## Figures and Tables

**Table 3:** Pivotal Trials for Management of Regional Nodes.

	Intervention	Population	MSS	DFS
MSLT-I Morton et al., 2014 [35]	WLE + SNB + CLND for positive nodes vs. WLE + SNB + nodal Observation Median Follow up: 10 years Primary Endpoint: MSS	Thick melanoma (3.5 mm) SLNB: 185 OBS: 126 Intermediate melanoma (1.2–3.5 mm) SLNB: 805 OBS: 522	Thick melanoma 10-y MSS SNB: 58.9 +/− 4.0% OBS: 64.4 +/− 4.6% HR 1.12; *p* = 0.56 OS: not reported Intermediate melanoma 10-y MSS: SNB: 81.4 +/− 1.5% OBS: 78.3 +/− 2.0% HR 0.84; *p* = 0.18 OS: not reported	Thick melanoma 10-y DFS: SLNB: 50.7 +/− 4.0% OBS: 40.5 +/− 4.7% HR 0.70; *p* = 0.03 Intermediate melanoma 10-y DFS SLNB: 71.3 +/− 1.8% OBS: 64.7 +/− 2.3% HR 0.76; *p* = 0.01
MSLT-II Faries et al., 2017 [38]	CLND vs. OBS with nodal ultrasound. CLND for nodal disease Median follow up: 43 months Primary Endpoint: MSS	ITT analysis n = 1934 Per protocol n = 1755 CLND: 824 OBS: 931	OBS vs. CLND MSS HR 1.08 95% CI, 0.88–1.34; *p* = 0.42 DMFS Adjusted HR 1.10 95% CI, 0.92–1.31; *p* = 0.31	CLND: 68.6, 1.7% OBS: 63.6, 1.7% log-rank *p* = 0.05
DeCOG-SLT Leiter et al., 2016 [85]	CLND vs. OBS for SNB positive patients Median Follow Up: 35 months Primary Endpoint: DMFS	n = 484 CLND: 242 OBS: 241	OBS vs. CLND OS HR 1.02 90% CI, 0.68–1.52; *p* = 0.95 RFS HR 0.959 90% CI, 0.70–1.31; *p* = 0.83 DMFS HR 1.19 90% CI, 0.83–1.69; *p* = 0.43	Regional Recurrence CLND: 8% OBS: 15%

Abbreviations: DFS, disease-free survival; DMFS, distant metastasis-free survival; CLND, completion lymph node dissection; MSS, melanoma-specific survival; OBS, observation group; OS, overall survival; SNB, sentinel lymph node biopsy.

**Table 4:** Evolution of Adjuvant Therapy for Cutaneous Melanoma.

Trial	Treatment Arms	Population	Key Findings
ECOG EST 1684 Enrollment: 1984–1990 [118]	IFNα2b vs placebo	Stage IIB–III (AJCC 2nd ed) n = 287	Median Follow Up: 12.6 years Improved RFS and OS with adjuvant IFNα2b at 5 years 12-year OS not significant Significant adverse events and treatment toxicity
EORTC 18071 Enrollment: 2008–2011 [122]	Ipilimumab vs placebo	Stage IIIA–IIIC (AJCC 7th ed) n = 951	Median Follow up: 6.9 years Improved RFS and OS with adjuvant ipilimumab No crossover as ipilimumab was not widely available
COMBI-AD Enrollment: 2013–2014 [123,124]	dabrafenib + trametinib vs. placebo	Stage IIIA–IIIC (AJCC 7th ed) n = 870	Median Follow Up: 5 years Improved RFS and DMFS with adjuvant BRAF and MEK inhibitors
CheckMate 238 Enrollment: 2015 [125]	Nivolumab vs ipilimumab	Stage IIIB–IV (AJCC 7th ed) n = 906	Median Follow up: 4.25 years Improved RFS with adjuvant nivolumab, similar OS Ipilimumab was not widely available
KEYNOTE-054 Enrollment: 2015–2016 [126]	Pembrolizumab vs placebo	Stage IIIA–IIIC (AJCC 7th ed) n = 1019	Median Follow up: 3 years Improved RFS with adjuvant pembrolizumab Fewer survival events than expected
BRIM 8 Enrollment: 2016–2017 [125]	vemurafenib vs. placebo	Cohort 1: Stage IIC-IIIB Cohort 2: stage IIIC (AJCC 7th ed) n = 184	Median Follow up: 2.8 years Adjuvant vemurafenib significantly improved DFS in cohort 1 DFS endpoint not met in cohort 2
SWOG 1404 Enrollment: 2015–2018 [117]	Pembrolizumab vs. ipilimumab or IFNα2b	Stage IIIA–IIIC (AJCC 7th ed.) n = 1301	Median Follow up: 4 years Improved RFS, but not OS, with adjuvant pembrolizumab Underpowered for OS events
KEYNOTE-716 Enrollment: 2018–2020 [119]	Pembrolizumab vs placebo	Stage IIB–IIC (AJCC 8th ed.) n = 976	Median follow up: 1.5 years Improved RFS with adjuvant pembrolizumab Final survival data pending

Abbreviations: AJCC, American Joint Committee on Cancer; DFS, disease-free survival; DMFS, distant metastasis-free survival; CLND, completion lymph node dissection; MSS, melanoma-specific survival; OBS, observation group; OS, overall survival; SNB, sentinel lymph node biopsy.

## Data Availability

Not applicable.
